# Potential Morbidity Reduction for Lung Stereotactic Body Radiation Therapy Using Respiratory Gating

**DOI:** 10.3390/cancers13205092

**Published:** 2021-10-12

**Authors:** Kim Melanie Kraus, Cristoforo Simonetto, Pavel Kundrát, Vanessa Waitz, Kai Joachim Borm, Stephanie Elisabeth Combs

**Affiliations:** 1 School of Medicine and Klinikum Rechts der Isar, Department of Radiation Oncology, Technical University of Munich (TUM), 81675 Munich, Germany; Vanessa.Waitz@tum.de (V.W.); Kai.borm@mri.tum.de (K.J.B.); stephanie.combs@tum.de (S.E.C.); 2 Department of Radiation Sciences (DRS), Institute of Radiation Medicine (IRM), Helmholtz Zentrum München (HMGU), 85764 Neuherberg, Germany; cristoforo.simonetto@helmholtz-muenchen.de; 3 Department of Radiation Dosimetry, Nuclear Physics Institute of the Czech Academy of Sciences, 180 00 Prague, Czech Republic; kundrat@ujf.cas.cz; 4 Deutsches Konsortium für Translationale Krebsforschung (DKTK), Partner Site Munich, 80336 Munich, Germany

**Keywords:** lung cancer, motion management, radiotherapy treatment planning, SBRT, radiation toxicity

## Abstract

**Simple Summary:**

Lung stereotactic body radiotherapy (SBRT) is the standard of care for early-stage lung cancer and oligometastases. For SBRT, motion has to be considered to avoid misdosage. Respiratory phase gating, meaning to irradiate the target volume only in a predefined gating motion phase window, can be applied to mitigate motion-induced effects. The aim of this study was to exploit the clinical benefit of gating for lung SBRT. For the majority of 14 lung tumor patients and various gating windows, we could prove a reduced dose to normal tissue by gating simulation. A normal tissue complication probability (NTCP) model analysis revealed a major reduction of normal tissue toxicity for moderate gating window sizes. The most beneficial effect of gating was found for those patients with the highest prior toxicity risk. The presented results are useful for personalized risk assessment prior to treatment and may help to select patients and optimal gating windows.

**Abstract:**

We investigated the potential of respiratory gating to mitigate the motion-caused misdosage in lung stereotactic body radiotherapy (SBRT). For fourteen patients with lung tumors, we investigated treatment plans for a gating window (GW) including three breathing phases around the maximum exhalation phase, GW40–60. For a subset of six patients, we also assessed a preceding three-phase GW20–40 and six-phase GW20–70. We analyzed the target volume, lung, esophagus, and heart doses. Using normal tissue complication probability (NTCP) models, we estimated radiation pneumonitis and esophagitis risks. Compared to plans without gating, GW40–60 significantly reduced doses to organs at risk without impairing the tumor doses. On average, the mean lung dose decreased by 0.6 Gy (*p* < 0.001), treated lung V20Gy by 2.4% (*p* = 0.003), esophageal dose to 5cc by 2.0 Gy (*p* = 0.003), and maximum heart dose by 3.2 Gy (*p* = 0.009). The model-estimated mean risks of 11% for pneumonitis and 12% for esophagitis without gating decreased upon GW40–60 to 7% and 9%, respectively. For the highest-risk patient, gating reduced the pneumonitis risk from 43% to 32%. Gating is most beneficial for patients with high-toxicity risks. Pre-treatment toxicity risk assessment may help optimize patient selection for gating, as well as GW selection for individual patients.

## 1. Introduction

Stereotactic body radiation therapy (SBRT) is a well-established high-precision radiotherapy method. It represents a standard of care for treatment of early-stage lung tumors and pulmonary oligometastases [1,2,3,4,5,6,7,8]. With excellent local control rates of up to 97% [9] for treatment of early-stage lung cancer, it provides a noninvasive treatment alternative for patients not suited to undergoing surgery. 

For peripheral tumors, the risk of adverse events is limited [10]. Depending on the tumor size and location, as well as the dose fractionation scheme, symptomatic radiation pneumonitis has been reported to occur in 10–20% of patients after SBRT [11,12,13,14]. For more critical tumor locations such as central (a location less than 2 cm from the proximal bronchial tree) or ultracentral lesions (overlap with the bronchial tree), the toxicity for the organs at risk (OARs) is increased [15,16,17,18]. The risk of high-grade esophagus toxicity for central tumor SBRT has recently been found to be low for moderate SBRT fractionation schemes [19]. A common solution to reduce toxicity for more ambitious dose regimes often is an adaptation of the dose regimes to meet the constraints for the OARs, leading to a tradeoff between local tumor control and toxicity [20,21,22,23]. 

When treating lung tumors with ablative doses, tumor motion has to be considered to avoid misdosage including both tumor underdosage and high-dose exposure of normal tissues. Commonly, this is addressed by the internal target volume (ITV) concept, in which the gross tumor volume (GTV) is delineated on each motion phase of 4D computed tomography (CT) and merged to an ITV covering the tumor in all motion states. In another method called respiratory gating, the treatment beam is switched off when the tumor moves out of a predefined motion window. Several studies revealed a comparable efficacy of the gating concept to other motion mitigation methods [24,25]. 

However, the performance of gating relies on the reproducibility of the breathing cycle and the extent of tumor motion. Another drawback of gating is the increased treatment time [26,27,28] depending on the duty cycle. Although in principle advantageous for all patients, one may expect gating to be especially beneficial in patients with critical scenarios such as central tumors or potentially increased toxicity risk in combination with systemic medication.

An estimation of the toxicity risk, the expected benefit from gating, and the selection of the optimal GW on a patient-specific basis would help fully exploit the potential of gating and overcome unnecessary treatment time prolongation. 

In this work, we assessed patient-specific dosimetric changes due to gating. We present a normal tissue complication probability (NTCP) model-based toxicity prediction on an individual patient basis and discuss the expected toxicity risk reduction achievable with gating in lung SBRT.

## 2. Materials and Methods

### 2.1. Patient Data

We investigated 14 patients, 4 with peripherally located tumors and 10 with tumors close to critical central organs. All patients suffered from early-stage lung cancer (T1a to T2c, 8th edition of TNM staging [29]) or lung metastases. The patient age ranged from 50 to 83 y, with a mean of 69.6 y. Twelve out of fourteen patients were current or former smokers. Seven patients suffered from pulmonary comorbidity, such as chronic obstructive pulmonary disease and fibrotic changes to the lung architecture. The patient characteristics are summarized in Table 1.

### 2.2. Treatment Planning

For all patients, we calculated volumetric modulated arc therapy (VMAT) treatment plans with variable gantry rotations for 6 MeV or 15 MeV photons based on 4D computed tomography (4DCT) acquired on a Somatom Emotion 16 CT (Siemens Healthineers, Erlangen, Germany). We identified 10 respiratory phases from 0 to 100, indicated in percent of the breathing cycle, where 50 denotes the maximum exhalation phase. For treatment plan calculation, we used the Eclipse v15.6 treatment planning software (Varian Medical Systems, Palo Alto, CA, USA). The GTV was delineated on a planning CT during free respiration and on all phase CT images. An ITV was defined that comprises the GTV in all respiratory phases for a standard treatment plan without gating and for a specified number of phases for the gating simulation. A planning target volume (PTV) was defined by adding an additional uniform margin of 5 mm to the ITV. The lungs, heart, and esophagus were delineated on the corresponding planning CT for all patient cases. Depending on the tumor location and clinical treatment plan, also the trachea, large bronchi, large vessels, chest wall, brachial plexus, spinal cord, larynx, and liver were delineated for some patients. For comparability, the simulated dose of 45 Gy delivered in 3 fractions was prescribed to the 65% isodose covering 98% or 99% of the PTV depending on the required constraints for all tumors regardless of the peripheral or central location. Dose constraints to the investigated OARs were assigned according to [5] for a three-fraction SBRT treatment procedure.

### 2.3. Respiratory Gating Simulation

A detailed description of the respiratory phase gating simulation method was published previously [30]. Treatment plans were copied on a specified number of respiratory phases defined as the gating window (GW), and the recalculated phase dose was transferred to a base phase that was closest to the maximum inhalation using B-spline multistage deformable image registration provided by the open-source image registration framework Plastimatch (www.plastimatch.org, accessed on 1 August 2019). On this base phase CT, the dose was accumulated for all phases of the GW. For comparison, the same procedure was applied to the standard treatment plan for all phases simulating the resulting doses without gating. Clinically relevant dose parameters such as the mean dose to the total lung minus GTV, the mean dose to the treated lung, the treated lung volume receiving 20 Gy, maximum doses to the esophagus and the heart, mean heart dose, and the dose to 5 cc of the esophagus were extracted according to the suggested guidelines provided in [5,30]. Here, we intentionally included not only the parameters used in the present toxicity risk analysis, but also further parameters often reported in the literature and/or in alternative risk models. To address the effect of gating on the treated tumor, doses to 2%, 50%, and 95% of the PTV were analyzed.

For all patients, a scenario was used that took a GW of 3 respiratory phases centered on the maximum exhalation (GW40–60), as this has been found to be the most stable in terms of tumor position (thus, gating was expected to be most efficient), and compared it to no gating [31,32,33]. For the comparison of different GWs, scenarios with an alternative 3-phase GW, comprising phases 20–40 at the rather steep part of the breathing cycle, and a large GW comprising 6 phases, GW20–70 were generated for a subset of 6 patients and compared to dose distributions without gating. GWs were chosen to be different in order to explore a higher diversity of effects. Patient-specific dosimetric changes were analyzed.

### 2.4. Statistical Analysis

To detect deviations from normality, the distributions over the patient group of dosimetric parameters and of the differences between plans without and with gating were tested using Kolmogorov–Smirnov tests (with a level of significance *p* = 0.05). After passing these tests, paired-sample ***t***-tests were applied to investigate differences between plans. To depict dose metric differences between plans, boxplots were used showing the medians, interquartile ranges, and extreme values. All analyses were performed with MATLAB R2020a (The MathWorks Inc., Natick, Massachusetts, MA, USA). 

### 2.5. Toxicity Risk Analysis

Following Ryckman et al. [34], symptomatic radiation pneumonitis risks after lung SBRT were calculated by a logistic function of the mean dose to the total lung minus the GTV. This logistic function is parameterized by TD50, the dose leading to a 50% risk, of 7.1 Gy, and by the normalized slope at the 50% response level, γ50 = 1.55. This risk model was derived from SBRT patients who received in most cases 48 Gy in 4 fractions or 50 Gy in 5 fractions. In the absence of more closely related risk estimates, this model was assumed applicable to the present study as well, with 45 Gy in 3 fractions.

Similarly, the risk for grade ≥2 esophagitis after lung SBRT was calculated by a logistic function of esophagus D5cc, converted to biologically effective dose BED10, which is delivered by a particular combination of the fraction dose and total dose using a tissue-specific α/β ratio of 10 Gy. The model parameters, TD50 = 39.82 Gy and γ50 = 1.0399, were taken from an analysis of the data from Wu et al. [35] reported by Nuyttens et al. [36].

## 3. Results

As expected, upon the use of gating, there was a general trend of dose reduction for OARs without impairment of PTV dose distributions. Clinical dose constraints could not be kept for all cases and for the investigated OARs since a very ambitious dose prescription scheme was chosen for all tumors, peripheral and central. Due to the very close proximity to the esophagus, esophageal dose constraints for Patient 7 were exceeded. For Patients 5, 7, 8, 10, and 13, the maximal heart dose was also high. 

**Figure 1:**
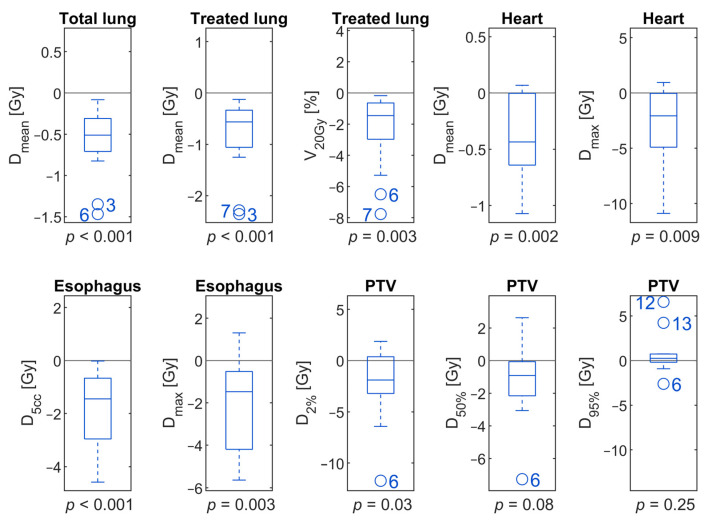
Boxplots showing the distribution of individual differences between the application of a GW40–60 compared to no gating. Boxes show interquartile ranges with the median denoted by a separating line. Outliers are marked by circles and patient case numbers.

Differences in dosimetric parameters between GW40–60 and no gating are listed in Table 2 and graphically depicted in Figure 1. There were significant reductions in the exposure of OARs in all dosimetric parameters investigated. Data for individual patients is given in Table S1.

The mean dose to the total lung was reduced by 0.6 Gy on average (*p* < 0.001) and by 0.8 Gy (*p* < 0.001) for the treated lung. V20Gy of the treated lung was reduced by 2.4% (*p* = 0.003). For Patient 7, treated lung Dmean and V20Gy were even reduced by 2.29 Gy and 7.77%.

The mean heart dose was reduced on average from 2.1 Gy without gating to 1.7 Gy with the application of GW40–60 (*p* = 0.002). Dmax was even reduced by 3.2 Gy on average (*p* = 0.009). However, dose reductions varied widely across individuals. For a single patient (Case 10), a reduction of the maximal dose to the heart of more than 10 Gy was achieved. On the other hand, there was another patient (Case 1) for whom Dmax was even increased by 1 Gy when gating was applied.

D5cc and Dmax of the esophagus dose were reduced by 1.8 Gy (*p* < 0.001) and 2.0 Gy on average by gating (*p* = 0.003). 

Dose distributions of the PTV were similar with and without gating applying GW40–60. However, D2% was significantly reduced for gating, on average by 2.2 Gy (*p* = 0.03).

**Figure 2:**
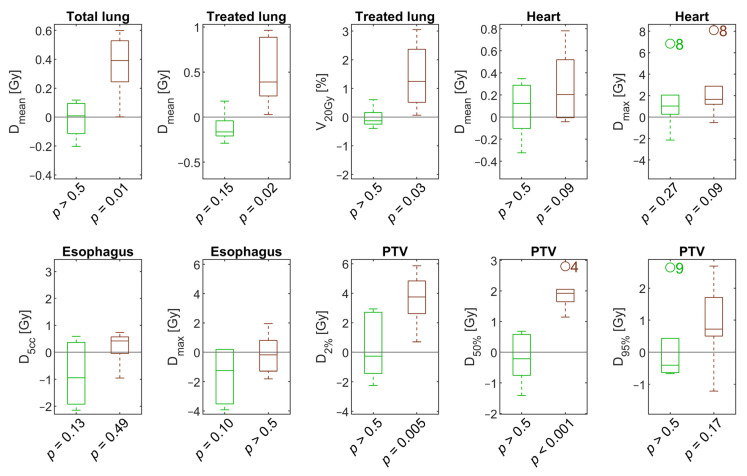
Boxplots showing the distribution of individual differences among the application of different gating windows. In each panel, the left part depicted in green represents the difference between plans with a gating window of 20–40 compared to 40–60. The right, brown part corresponds to the difference between a gating window of 20–70 and 40–60. Boxes show interquartile ranges with the median denoted by a separating line. Outliers are marked by circles and patient case numbers.

Comparing different GWs for a subset of patients, we found differences less pronounced and not statistically significant when GW20–40 was compared to GW40–60, as depicted with green boxes in Figure 2. D5cc and Dmax of the esophagus were reduced by 0.8 Gy (*p* = 0.13) and 1.6 Gy (*p* = 0.10) on average when a GW of 20–40 was applied. Differences in the mean doses to the total or treated lung were below 0.3 Gy for all patients. 

More interestingly, we noticed major dosimetric differences for GW20–70 compared to GW40–60 (brown boxes, Figure 2). In almost all investigated scenarios, the dosimetric parameters were worse compared to GW40–60. On average, the mean total lung dose increased by 0.4 Gy (*p* = 0.01) and V20Gy of the treated lung by 1.4% (*p* = 0.03) for GW20–70. The mean and maximum heart doses increased by 0.3 Gy and 2.5 Gy, but the results were not significant. The maximum esophagus dose even slightly decreased for GW20–70 compared with GW40–60.

**Figure 3:**
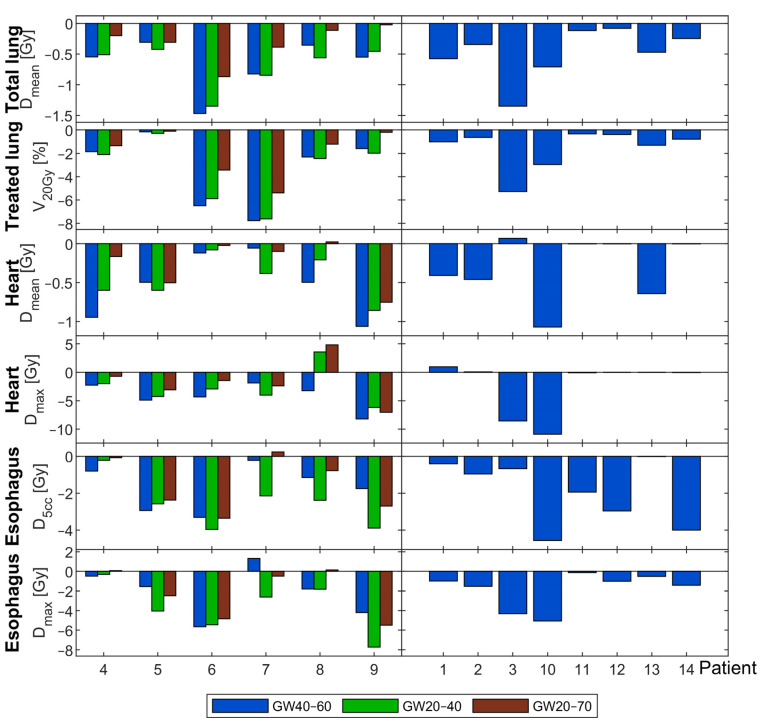
**Left**: Changes in dosimetric parameters for different GWs for a subset of 6 patients. Blue bars refer to the differences between GW40–60 compared to no gating; green bars refer to differences between GW20–40 compared to no gating; brown bars refer to the differences between GW20–70 to no gating. **Right**: Dosimetric changes between GW40–60 and no gating for the residual 8 patients.

Analyzing the results for different GWs on a patient-specific level, no clear trend could be found comparing the reductions by gating with GW20–40 or GW40–60 relative to plans without gating, as indicated in Figure 3. The largest individual changes of Dmean for the total lung of 1.47 Gy and 1.35 Gy for GW40–60 and GW20–40 were noticed for Patient 6. The largest reductions in V20Gy for the treated lung of 7.77% for GW40–60 and 7.61% for GW20–40 were obtained for Patient 7. However, for the same Patient 7, GW20–40 reduced Dmax and D5cc of the esophagus by 2.61 Gy and 2.15 Gy, while GW40–60 increased esophagus Dmax by 1.31 Gy and reduced its D5cc by 0.23 Gy relative to plans without gating.

The expected trend that a larger GW increases doses to OARs became obvious for GW20–70, which in general resulted in smaller reductions of doses with respect to the plans without gating (brown bars in Figure 3) than what was achieved with GW40–60 (blue bars) and/or GW20–40 (green bars). However, for Patient 9, the maximum esophagus dose was reduced from the plan without gating by 5.49 Gy upon the larger GW20–70, more than upon GW40–60, which spared 4.2 Gy.

**Figure 4:**
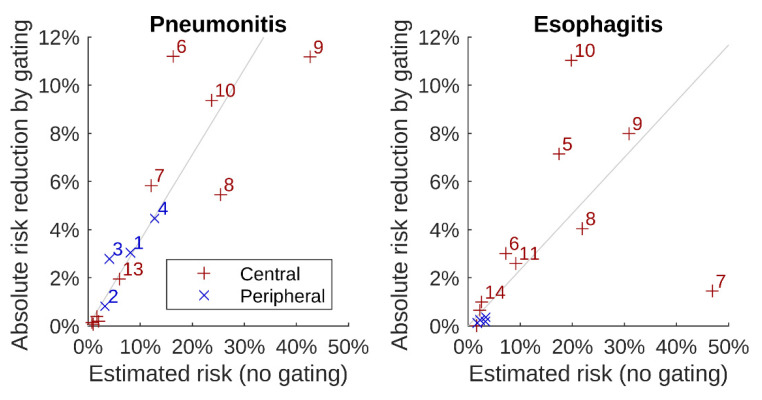
Absolute reduction in estimated radiation pneumonitis risk (**left**) and esophagitis risk (**right**) as a function of estimated risk of SBRT without gating. For the gating plans, a window of 40–60 was applied. Each symbol represents one patient case as indicated with numbers next to the symbol. Red color (+ symbols) indicates central tumors, and blue color (× symbols) indicates peripheral tumors. Grey lines indicate the mean risk reductions of 36% for pneumonitis and 23% for esophagitis.

When symptomatic radiation pneumonitis risks were calculated by a logistic function of mean total lung dose, we found that the mean estimated risk for radiation pneumonitis was 11% without gating (Table 2). Application of gating with a GW40–60 reduced it to 7% (relative reduction by 36%). For esophagitis, the mean estimated risk without gating was 12%. This figure decreased to 9% upon GW40–60 (relative reduction by 23%). Patient-specific estimated risks and their reductions upon gating are shown in Figure 4. As can be seen, major reductions in toxicity risks were achieved for patients with higher toxicity risks without gating. However, patient individual risks majorly differed. For Patient Case 9, the estimated pneumonitis risk was 43%, which led to an absolute pneumonitis risk reduction of 11% for gating with GW 40–60, whereas for the five patients with estimated toxicity risks below 4%, the absolute risk reduction by gating was below 1%. For Patient Case 7 with the absolute highest D5cc, the estimated esophagitis risk was 47%, which was not clearly reduced by gating.

## 4. Discussion

Individual patient risk for toxicity in the context of motion-guided radiotherapy is essential to ensure optimal dose coverage and minimal side effects. We analyzed the effect of gating on dose to normal tissue and calculated the corresponding individual risk for side effects for each organ. We could demonstrate that especially for patients with the highest risks for toxicity, gating was shown to be an effective mitigation measure in an NTCP model-based risk analysis.

On average, doses to the lung and esophagus were reduced for all GWs without a major impact on PTV doses. Clearly, absolute dose values of the present study have to be evaluated with caution, since clinically acceptable dose constraints could not be kept in all cases due to the proximity to the OAR and a very ambitious dose regime. The dose and fractionation scheme might not be applicable for all tumor locations, especially for central tumors. Nevertheless, for interindividual comparability and in order to show the potential dose reductions by gating, we consistently used the 45 Gy in three fractions scheme in the present “proof-of-principle” study. Clearly, there is currently no application for such a dose regime where the tumor is in close vicinity to relevant OARs, and severe side effects are expected. Furthermore, the fixed prescription scheme might limit the potential effect of gating that might be reached for specific patients and other dose regimes. The general trend of dose reduction by gating has been shown before [25,28,35]. Prunaretty et al. showed a reduction of V20Gy by 33% [25]. Rouabhi et al. [28] reported a reduction of the mean lung dose between 16.1% and 6.0% and of V20Gy between 20.0% to 7.2%. However, the clinical benefit of gating was questioned by others, who found only small dosimetric impact by gating [37,38] and thus concluded gating to be a solution only for a well-selected group of patients [25]. 

Our analysis revealed that an increased estimated toxicity risk is connected with an increased risk reduction for pulmonary or esophageal toxicity by gating in lung SBRT. This leads to the assumption that gating is most beneficial for those patients who would most likely suffer from radiation-induced toxicity. We estimated a mean pneumonitis risk of about 11% without gating, in agreement with the literature [11,12,13,14]. A GW around the exhalation phase, commonly considered reasonable [36,38], led to relative lung toxicity reductions of 36% on average. The mean esophageal toxicity risk was reduced by up to 23% by gating.

When comparing different GW sizes, we found that massively increasing the GW including six breathing phases (GW20–70) compared to three phases (GW40–60) impaired almost all investigated dose parameters for all scenarios. Jang et al. [39] also investigated the effects of different GW sizes and found smaller GWs with a duty cycle of 25%, leading to small dosimetric improvements for individual patients for whom a duty cycle of 50% did not lead to adequate motion mitigation. Other factors such as tumor motion range in the anterior–posterior direction were also found to be predictive for the dosimetric benefit of gating. Despite the general trend of a larger dose reduction for smaller GW sizes, we found for individual patients and OARs favorable dose characteristics even for the largest investigated GW20–70. For one patient (Patient 9), esophageal dose was reduced more by a larger GW than by a smaller one. On the first view, these findings were unexpected; however, one has to be aware that the gating window is based on the breathing curve, which might not necessarily correlate directly with the movement direction and extension of other organs. In a previous work, we analyzed tumor breathing motion [40] and revealed that individual anatomical aspects might be of similar relevance. These findings suggest that risk estimation and evaluation of the optimal GW for individual patients might be needed. Whereas for patients with prior interstitial lung disease, the pneumonitis risk should be kept as low as possible, others might profit from a reduced esophageal dose, e.g., in the case of thoracic re-irradiation. 

The model-based risk analysis revealed individual patients for whom the risk of esophageal toxicity was not clearly reduced by gating with GW40–60. This effect is most likely caused by patient individual tumor location in relation to OARs and the extent of breathing motion. For Patient 7, for instance, for whom we found a high esophagitis risk, the tumor was in very close vicinity to the esophagus. For GW20–40, sparing of the esophagus was better, but the difference from GW40–60 was not significant.

Clearly, our study was limited by a rather small number of patients; however, multiple and diverse gating scenarios were analyzed. The analysis was based on a 4D CT and deformable image registration where image quality and the irregularity of the breathing cycle might affect the dosimetric results. Deformable image registration is still challenging, especially in the case of strong and opposing tissue movements. Error estimation such as often performed with similarity measures is also challenging [41]. In this study, however, we visually assessed each registration by an experienced physicist and physician in order to ensure valid registration results. Furthermore, the method of dose accumulation over the breathing cycle used here seems to represent a valid approach that is close to reality. Of course, gating relies on the reproducibility of the breathing cycle. Breath hold techniques that have proven to improve the tumor location reproducibility [42,43] might further improve the results presented here.

Our results support the approach by an individual risk calculation helping to select the appropriate patients and gating window in contrast to the use of a standard ITV concept and standard margins [44]. 

## 5. Conclusions

Gating has the potential to reduce lung and esophageal toxicity for lung SBRT. For patients with the highest risks for toxicity, gating was revealed to be most effective in an NTCP model-based risk analysis. Thus, gating could be especially useful for tumors in close locations to OARs such as centrally located tumors, for patients with pulmonary comorbidities or previous thoracic radiotherapy. In order to find the best tradeoff between dose and treatment time, our results suggest an individual patient pretreatment toxicity risk analysis based on the calculated treatment plan in order to facilitate efficient patient selection for gating and the choice of the optimal gating window.

## Figures and Tables

**Table 1:** Patient characteristics.

Case	Case Location Peripheral	Age	Smoking Status	TNM	Pulmonary Comorbidity
1	Right lower lobe	81	Current	cT1c cN0 M0	No
2	Left lower lobe	72	Former	cT1c cN0 cM0	Yes
3	Left upper lobe	79	Former	cT1 cN0 cM0	Yes
4	Right lower lobe	83	Former	cT1b cN0 M0	Yes
	**Case Location Central**				
5	Right hilar	74	Current	cT1b2 cN0 M0	Yes
6	Left hilar	63	Former	cT1c cN0 cM0	Yes
7	Right hilar	80	Former	pT2a pN0 cM0	Yes
8	Left hilar	83	Former	rcT0 rcN1 rcM1	No
9	Right hilar	50	Former	cT4 cN+ cM1	No
10	Right hilar	55	Current	cT2 cN2 M1b	No
11	Left hilar	70	Former	cT2 cN0 cMX	Yes
12	Left hilar	66	Current	cT3 cN1 cM1	No
13	Right hilar	70	no	rcT1a pN1 M0	No
14	Right upper lobe	51	no	cT1b cN1 cM1b	No

**Table 2:** Differences in dosimetric parameters and in the risks of pneumonitis and esophagitis between gating window GW40–60 and no gating. Reported are the mean values and standard deviations (std. dev.) over the 14 studied patients.

Dosimetric Parameter	Gating Window GW40–60		No Gating	
	Mean	Std. Dev.	Mean	Std. Dev.
Total lung D_mean_ (Gy)	3.5	1.5	4.1	1.6
Treated lung D_mean_ (Gy)	5.8	2.7	6.6	3.0
Treated lung V_20Gy_ (%)	8.0	5.0	10.4	6.7
Heart D_mean_ (Gy)	1.7	1.4	2.1	1.8
Heart D_max_ (Gy)	16.0	15.0	19.2	16.2
Esophagus D_5cc_ (Gy)	8.3	7.2	10.1	7.3
Esophagus D_max_ (Gy)	18.8	12.0	20.8	11.6
PTV D_2%_ (Gy)	64.3	2.9	66.5	5.0
PTV D_50%_ (Gy)	54.1	1.5	55.3	3.2
PTV D_95%_ (Gy)	45.1	1.0	44.4	2.5
Pneumonitis risk (%)	7.4	8.9	11.4	12.1
Esophagitis risk (%)	9.3	12.3	12.2	13.7

## Data Availability

The data presented in this study are available in this article and Supplement Table S1.

## Supplementary Materials

The following are available online at https://www.mdpi.com/article/10.3390/cancers13205092/s1, Table S1: Dosimetric data with and without gating.
