# Supplementary material for: Potential Morbidity Reduction for Lung Stereotactic Body Radiation Therapy Using Respiratory Gating
Source: Cancers (Basel). 2021 Oct 12;13(20):5092. doi: 10.3390/cancers13205092 (PMC8533802; doi:10.3390/cancers13205092)
Supplement: Supplementary file 1 [file cancers-13-05092-s001.zip › cancers-1396969-supplementary.pdf]

Table S1. Dosimetric data with and without gating.

Case	GW [phase]	PTV [cm³]	PTV			Lung			Esophagus		Heart		Trachea/ large bronchii	Aorta	Spinal Cord	Ribs/ chest wall
			D_2 [Gy]	D_{50} [Gy]	D_{95} [Gy]	D_{mean} (Total Lung-GTV) [Gy]	V_{20Gy} (lung treated) [%]	D_{mean} (lung treated) [Gy]	D_{max} [Gy]	D_{5cc} [Gy]	D_{max} [Gy]	D_{mean} [Gy]	D_{max} [Gy]	D_{max} [Gy]	D_{max} [Gy]	D_{max} [Gy]
1	40–60	43.3	61.2	51.6	45.2	3.75	6.5	5.9	9.0	5.8	13.8	2.3	14.2	8.3	12.3	49.5
	no gating	59.5	62.2	51.4	45.0	4.32	7.5	6.5	10.0	6.2	12.9	2.7		8.7	17.1	56.6
2	40–60	35.0	67.6	56.0	46.0	2.86	5.1	4.2	14.1	2.5	13.3	2.7		8.3	12.3	49.5
	no gating	31.7	70.3	56.1	45.3	3.21	5.7	4.6	15.7	3.4	13.2	3.2		27.0	18.2	52.9
3	40–60	11.6	62.4	53.1	45.3	2.13	4.2	3.9	6.9	0.6	4.6	0.3		11.8	5.5	
	no gating	34.1	62.5	55.1	44.6	3.48	9.5	6.3	11.2	1.2	13.2	0.2	9.4	15.6	7.3	
4	40–60	48.8	66.4	55.1	43.1	4.35	8.2	7.1	8.1	5.6	12.0	4.5		7.2	9.6	
	20–40	59.7	69.1	55.8	42.5	4.39	8.0	7.1	8.3	6.2	12.3	4.8	0.3			59.1
	20–70	59.3	72.3	57.9	41.9	4.70	8.8	7.5	8.7	6.3	13.6	5.2	0.3		10.5	61.7
	no gating	79.8	69.6	56.4	42.9	4.90	10.1	8.2	8.6	6.4	14.3	5.4	0.5		10.6	58.6
5	40–60	14.9	68.7	56.7	46.0	2.14	2.0	2.8	28.1	13.3	22.6	1.0		14.6	17.4	
	20–40	15.4	68.0	56.0	45.4	2.03	1.9	2.7	25.7	13.6	23.2	0.9		14.2	18.0	
	20–70	15.9	73.5	58.4	46.8	2.14	2.1	2.8	27.2	13.8	24.4	0.9		13.9	19.0	
	no gating	17.3	70.6	58.9	45.9	2.45	2.2	3.1	29.7	16.2	27.5	1.5	65.6	16.1	18.5	
6	40–60	36.1	67.8	56.0	45.8	3.76	11.0	6.2	11.6	7.9	4.2	0.3		28.9	7.6	
	20–40	38.1	65.6	54.5	45.1	3.88	11.6	6.4	11.7	7.3	5.7	0.3		27.9	6.1	
	20–70	42.2	68.5	57.8	48.5	4.36	14.1	7.2	12.4	7.9	7.1	0.4		30.3	7.3	
	no gating	48.3	79.5	63.2	48.4	5.23	17.5	6.5	17.2	11.2	8.6	0.4	17.6	38.5	11.6	28.4
7	40–60	28.2	61.2	54.0	46.1	4.01	15.1	9.8	48.0	22.0	34.7	2.4	13.8		13.9	24.8
	20–40	29.4	64.1	54.6	45.9	3.98	15.3	9.6	44.1	20.0	32.6	2.1	10.7		16.5	23.1
	20–70	34.7	65.8	56.1	46.7	4.44	17.5	10.7	46.2	22.4	34.2	2.4	13.3		17.3	26.8
	no gating	41.8	67.6	55.5	46.3	4.83	22.9	12.1	46.7	22.2	36.6	2.5	20.1	16.7	21.9	26.5
8	40–60	83.1	60.9	52.9	45.1	5.51	16.7	8.9	29.2	16.3	48.7	2.5		56.6	12.4	
	20–40	80.4	61.1	52.7	45.5	5.31	16.6	8.6	29.2	15.1	55.6	2.8		57.4	10.7	
	20–70	89.9	63.5	55.0	46.8	5.75	17.8	9.1	31.1	16.7	56.8	3.1		58.4	13.0	
	no gating	99.2	64.4	56.0	46.0	5.87	19.0	9.6	31.0	17.5	52.0	3.0	42.5	60.3	13.8	
9	40–60	71.1	62.6	54.3	43.2	6.21	12.9	9.2	28.4	17.7	10.8	2.2		26.9	15.9	
	20–40	61.7	61.2	54.1	45.9	6.30	12.5	9.0	24.9	15.6	12.9	2.4		23.3	18.7	
	20–70	75.7	65.5	55.5	43.7	6.73	14.3	9.6	27.1	16.8	12.0	2.5		26.1	20.1	
	no gating	91.6	65.1	56.5	43.8	6.76	14.5	9.7	32.6	19.5	19.0	3.2		29.7	18.0	
10	40–60	62.9	59.9	51.8	44.9	5.05	14.2	9.0	20.5	12.3	33.5	3.3		22.9	14.4	
	no gating	93.5	59.1	52.0	44.1	5.76	17.2	10.2	25.6	16.9	44.4	4.3		28.6	16.4	
11	40–60	40.7	64.5	54.4	45.9	2.53	4.2	3.3	19.0	10.6	0.8	0.1		49.6	13.2	
	no gating	45.0	66.4	54.9	45.6	2.65	4.5	3.6	19.1	12.6	0.9	0.1		55.7	10.8	
12	40–60	16.4	64.2	53.3	45.3	1.72	3.7	2.9	25.9	0.4	0.1	0.0		53.8	7.8	
	no gating	18.2	62.3	50.7	38.7	1.81	4.1	3.1	26.9	3.4	0.2	0.0		48.3	7.8	
13	40–60	8.9	65.6	53.5	44.3	3.48	6.6	6.2	6.8	0.5	25.3	2.7		5.4	4.1	
	no gating	14.0	65.1	52.3	40.1	3.95	7.9	6.9	7.3	0.6	25.3	3.3	0.6	5.9	4.0	
14	40–60	8.5	67.0	55.2	45.3	1.26	2.2	2.0	8.2	0.5	0.1	0.0		10.0	6.6	
	no gating	12.2	66.6	55.5	44.6	1.51	3.0	2.5	9.6	4.5	0.2	0.0	12.0	12.0	8.5	
